# Functional analysis of splicing mutations in exon 7 of *NF1 *gene

**DOI:** 10.1186/1471-2350-8-4

**Published:** 2007-02-12

**Authors:** Irene Bottillo, Alessandro De Luca, Annalisa Schirinzi, Valentina Guida, Isabella Torrente, Stefano Calvieri, Cristina Gervasini, Lidia Larizza, Antonio Pizzuti, Bruno Dallapiccola

**Affiliations:** 1 IRCCS-CSS, San Giovanni Rotondo and CSS-Mendel Institute, Rome, Italy; 2 Department of Experimental Medicine and Pathology, University of Rome "La Sapienza", Rome, Italy; 3 Department of Dermatology-Venereology and Plastic and Reconstructive Surgery, University of Rome "La Sapienza", Rome, Italy; 4 Division of Medical Genetics, San Paolo School of Medicine, University of Milan, Milan, Italy

## Abstract

**Background:**

Neurofibromatosis type 1 is one of the most common autosomal dominant disorders, affecting about 1:3,500 individuals. *NF1 *exon 7 displays weakly defined exon-intron boundaries, and is particularly prone to missplicing.

**Methods:**

In this study we investigated the expression of exon 7 transcripts using bioinformatic identification of splicing regulatory sequences, and functional minigene analysis of four sequence changes [c.910C>T (R304X), c.945G>A/c.946C>A (Q315Q/L316M), c.1005T>C (N335N)] identified in exon 7 of three different *NF1 *patients.

**Results:**

Our results detected the presence of three exonic splicing enhancers (ESEs) and one putative exonic splicing silencer (ESS) element. The wild type minigene assay resulted in three alternative isoforms, including a transcript lacking *NF1 *exon 7 (NF1ΔE7). Both the wild type and the mutated constructs shared NF1ΔE7 in addition to the complete messenger, but displayed a different ratio between the two transcripts. In the presence of R304X and Q315Q/L316M mutations, the relative proportion between the different isoforms is shifted toward the expression of NF1ΔE7, while in the presence of N335N variant, the NF1ΔE7 expression is abolished.

**Conclusion:**

In conclusion, it appears mandatory to investigate the role of each nucleotide change within the *NF1 *coding sequence, since a significant proportion of *NF1 *exon 7 mutations affects pre-mRNA splicing, by disrupting exonic splicing motifs and modifying the delicate balance between aberrantly and correctly spliced transcripts.

## Background

Alternative splicing, the process by which exons are included or excluded in the mature mRNA, is an important mechanism whereby different transcripts are generated from the same gene unit. In fact, most human genes are transcribed in multiple alternative mRNAs, according to different regulatory programs, resulting in functionally different protein isoforms [1]. In the best characterized models of vertebrate cell-specific alternative splicing, post-transcriptional regulation is tissue or developmental stage specific and may be mediated by intronic and exonic cis elements. These elements, which are important for the correct splice-site identification, can act by stimulating (exonic splicing enhancers, ESEs) or repressing (exonic splicing silencers, ESSs) the exon's splicing [2].

Neurofibromatosis type 1 (NF1, MIM#162200) is one of the most common autosomal dominant disorders, affecting about 1:3,500 individuals in all ethnic groups. The *NF1 *gene is approximately 280 kb in size and maps to chromosome 17q11.2 [3-5]. *NF1 *contains 60 exons, with an 11- to 13-kb transcript and an open reading frame coding for 2,818 amino acids [6]. The disease is fully penetrant and the diagnosis of NF1 is based on the clinical criteria recommended by NIH Consensus Conference (Stumpf, et al., 1988), which include multiple cafè-au-lait spots, cutaneous or subcutaneous neurofibromas, plexiform neurofibromas, axillary or inguinal freckling, optic gliomas, and iris Lish nodules. Although *NF1 *mutations are distributed along the entire coding sequence, no genotype-phenotype correlation has been found so far [7], with the exception of the recurrent and atypical deletions underlying NF1 microdeletion syndrome [8].

The *NF1 *gene is ubiquitously expressed, and four normal in-frame *NF1 *splice isoforms are known, brain specific 9br isoform (30 bp) [9], exon 10a-2 isoform (45 bp) [10], exon 23a isoform (63 bp), which are expressed in all tissues at various levels [11], and the muscle specific exon 48a isoform (54 bp) [12]. In addition, several other alternative transcripts have been described, including ex29-, ex30-, ex29/30- and the N-isoform [13,14].

Mutation analysis has shown that approximately 50% of *NF1 *mutations result in splicing alterations [15-18]. In some cases, splicing mutations do not occur at the conserved AG/GT dinucleotides of the splice sites. For example, mutations leading to stop codons in exon 7 and 37 of *NF1 *gene have been reported to be involved in exon skipping [18-21]. In addition, mutational analysis of the *NF1 *gene disclosed several additional splice variants in which specific exons are skipped in fresh lymphocytes of unaffected persons, albeit typically at low levels [22,23]. A number of studies have also reported that some of these transcripts are more abundant when RNA from aged blood or from blood kept at non-physiological temperatures is analyzed [15,22,24,25].

The expression of an alternative transcript lacking exon 7 has been demonstrated [17,26]. Indeed, *NF1 *exon 7 displays weakly defined exon-intron boundaries, and is particularly prone to aberrant splicing. In the present study, we have used *in silico *and *in vitro *analysis to evaluate the functional consequences on gene expression of four nucleotide variants detected in *NF1 *exon 7, including a nonsense mutation (R304X), a missense mutation (L316M), and two silent changes (Q315Q and N335N). Since both Q315Q and L316M mutations were together in *cis *in the same patients [27], our analysis aim was to understand their effect together and independently.

## Methods

### DNA mutation analysis

**Figure 1:**
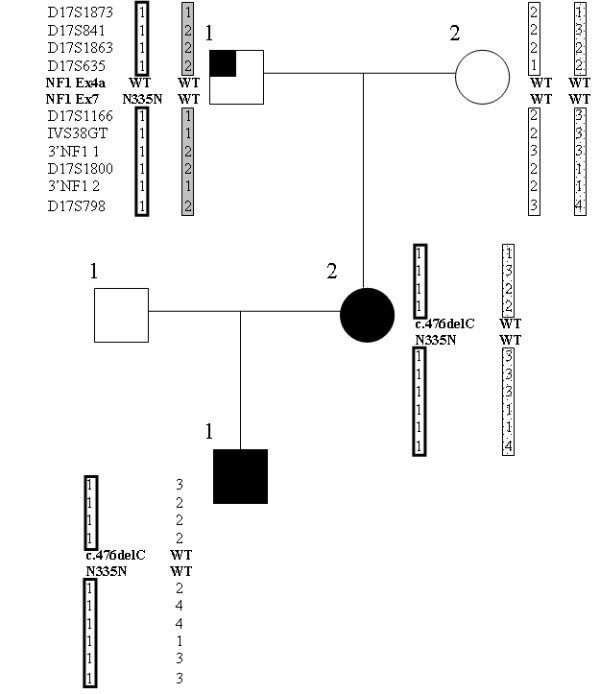
Family NF-01, showing pedigree numbers by generation and person number (i.e., I:1 to III:1). NF1 affected individuals are denoted by a blackened symbol, unaffected by an unblackened symbol. The individual carrying isolated neurofibromas is denoted by a partially blackened symbol. The haplotypes from linkage analysis of chromosome 17 are shown. Individuals II:2 and III:1 carry the N335N silent mutation and the c.476delC deletion, while individual I:1 carries only the N335N variant.

The nucleotide variants investigated in this study include a recurrent nonsense mutation [c.910C>T (R304X)] [22,27-29] and a novel silent change [c.1005T>C (N335N)] identified using denaturing high performance liquid chromatography (dHPLC) followed by bidirectional sequencing, as well as a silent change and a missense mutation occurring together *in cis *in the same patient [c.945G>A/c.946C>A (Q315Q/L316M)], and previously reported by us [27]. PCR conditions, amplicon length, and resolution temperatures for dHPLC analysis are reported elsewhere [28,30]. The N335N silent change was found in a two generation NF1 family (family NF-01) carrying another *NF1 *gene mutation, a frameshift deletion (c.476delC) in exon 4a. The silent change N335N was found in the proband (II-2) and her child (III-1), both affected by NF1, and in the proband's father (I-1) presenting out of NF1 clinical signs only three cutaneous neurofibromas. Frameshift mutation c.476delC was detected in the proband and her child, but not in the proband's father. Both N335N and c.476delC were not found in 200 healthy subjects. Microsatellite analysis performed using 10 markers tightly linked to the *NF1 *locus (D17S1873, D17S841, D17S1863, D17S635, D17S1166, IVS-38, 3'NF1-1, D17S1800, 3'NF1-2, D17S798) showed that N335N and c.476delC co-segregated on the same chromosome 17. Pedigree microsatellite analysis details of family NF-01 are shown in Figure 1. The project was approved by the institutional review board and all participants provided informed consent.

### Prediction of exonic splice enhancers

The *NF1 *exon 7 coding sequence including sequence changes Q315Q, L316M, R304X and N335N was analyzed using the online ESEfinder web interface (Release 1.1) [31] which performs searches for putative ESEs in query sequences using weight matrices corresponding to the motifs of four different human serine/arginine-residue proteins (SR proteins): SF2/ASF, SC35, SRp40, and SRp55. The score for a given sequence was considered to be potentially significant if greater than the default threshold value defined in the input page. These values were set as follows: 1.956 for SF2/ASF, 2.383 for SC35, 2.670 for SRp40, and 2.676 for the SRp55.

### Minigene construction and expression

Minigene constructs were generated and transfected into cos-1 cells. Exon 7 of the *NF1 *gene and flanking intronic sequence were amplified using as template the DNA of a non-affected individual. Primers NF1-7F (5'-cacacactcgagAACAGCTTGTTTGGGAAGGA-3') and NF1-7R (5'-cacacaggatccGGCCCTAATTGCCACATTATT-3') were used to generate a fragment containing exon 7 as well as 258 bp of 5' and 248 bp of 3' of intronic flanking sequence. The forward primer contains a 5'-XhoI linker, whereas the reverse primer contains a 5'-BamHI linker. The PCR amplification reaction was performed on 100 ng genomic DNA in a standard 50 μL volume, containing 1× Optimase reaction buffer (Transgenomic, Crewe, UK), 1.5 mM MgSO_4_, 1 μM of each primer, 0.2 μM dNTP_s_, and 2.5 U Optimase TaqDNA polymerase (Transgenomic, Crewe, UK) in a 9700 thermal cycler (Applied Biosystem, FosterCity, CA). Thermal conditions were 35 cycles of 95°C for 30 seconds, 57°C for 30 seconds, and 72°C for 1 minute, preceded by 5 minutes at 95°C and followed by a final elongation step at 72°C for 10 minutes.

**Figure 2:**
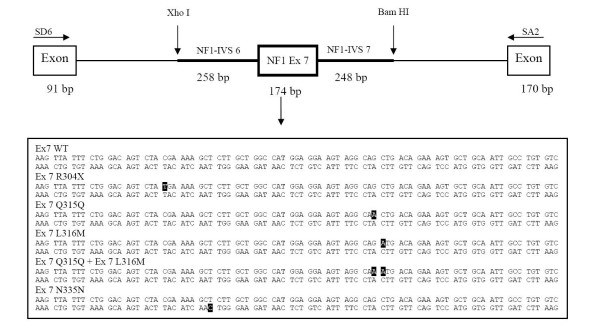
Representation of the pSPL3-NF1-7 minigene construct. Genomic sequence from exon 7 of the *NF1 *gene along with 258 bp of 5' and 248 bp of 3' intronic flanking sequence was ligated into pSPL3 plasmid. Shaded sequences describe the nucleotide changes introduced independently by *in vitro *mutagenesis to obtain the mutated constructs.

After confirmation of successful amplification through detection of the expected 680 bp band on the agarose gel, the products were digested with XhoI (Promega, Madison, WI, USA) and BamHI (Promega, Madison, WI, USA) restriction enzymes. The exon trapping expression vector pSPL3 (Invitrogen Corporation, Carlsbad, CA) contains a replicon and Ap^r ^marker for growth in *E. coli*, an SV40 segment for replication and transcription in cos-1 cells, HIV-1 tat splicing signals, and a multiple cloning site. The tat segment contains an intron, splice donor (SD) and splice acceptor (SA) sites, and flanking exon sequences. The insert was directly ligated between the SD and SA sites into the XhoI/BamHI restriction points. Ligation into pSPL3 was performed at room temperature for 30 min, using T4 DNA ligase (Invitrogen Corporation, Carlsbad, CA). *E. coli *DH5α competent cells (Invitrogen Corporation, Carlsbad, CA) were transformed with the plasmid constructs and plated overnight. The resulting clones were checked for fragment orientation and sequenced. Minigene constructs were isolated using a midiprep kit (Qiagen, Hilden, Germany). The resulting pSPL3-NF1-7-wt minigene construct is shown in Figure 2. The sequence changes were independently introduced in the pSPL3-NF1-7-wt by means of the QuickChange Site-Direct Mutagenesis Kit (Stratagene, La Jolla, CA), as instructed by the manufacturer. The changes made in pSPL3-NF1-7-wt are shown in Figure 2, and Table 1 lists the mutagenic primers used. All the mutants were sequenced to confirm that only the desired changes were introduced, and were then isolated with a miniprep kit (Qiagen, Hilden, Germany). The minigene constructs containing either the wild type sequence or a exon 7 variant were transfected into cos-1 cells by electroporation.

**Table 1:** Sequence primers used for *in vitro *mutagenesis.

**Mutation**	**Mutagenic primer plus**	**Mutagenic primer minus**
R304X	5'-AAGTTATTTCTGGACAGTCTATGAAAAGCTCTTGCTGGCCATGG-3'	5'-CCATGGCCAGCAAGAGCTTTTCATAGACTGTCCAGAAATAACTT-3'
Q315Q	5'-GGCCATGGAGGAAGTAGGCAACTGACAGAAAGTGCTGCAATTG-3'	5'-CAATTGCAGCACTTTCTGTCAGTTGCCTACTTCCTCCATGGCC-3'
L316M	5'-GGCCATGGAGGAAGTAGGCAGATGACAGAAAGTGCTGCAATTG-3'	5'-CAATTGCAGCACTTTCTGTCATCTGCCTACTTCCTCCATGGCC-3'
Q315Q+L316M	5'-GGCCATGGAGGAAGTAGGCAAATGACAGAAAGTGCTGCAATTG-3'	5'-CAATTGCAGCACTTTCTGTCATTTGCCTACTTCCTCCATGGCC-3'
N335N	5'-GTAAAGCAAGTACTTACATCAACTGGGAAGATAACTCTGTC-3'	5'-GACAGAGTTATCTTCCCAGTTGATGTAAGTACTTGCTTTAC-3'

Approximately 2.5 × 10^6 ^cos-1 cells were trypsinized, washed with cold PBS 1× buffer (Mg^2+ ^and Ca^2+ ^free) and then resuspended in 800 μl of the same PBS solution. Two μg of normal or mutant minigene DNAs were added to the cell suspension in 0.4 cm gap sterile cuvettes. The cell-DNA mixture was then incubated on ice for 10 min and electroporated at 4°C using a Bio-Rad Gene Pulser II (Bio-Rad, Richmond, CA) at a setting of 300 Volts, 250 μF. Electroporated cells were then incubated on ice for 10 min, diluted 1:20 in complete medium and cultured in T-75 flasks at 37°C in a humidified atmosphere under 5% CO_2 _for at least 48 h. Total cellular RNA from the transfected cos-1 cells was purified by the acid guanidine-phenol-chloroform method and used for RT-PCR to confirm the splicing patterns. First strand cDNA was synthesized from 2 to 3 μg of total RNA by random-primed reverse transcription with Superscript II Reverse Transcriptase (Invitrogen Corporation, Carlsbad, CA). To evaluate the pattern of transcripts from the transfected minigenes, the following vector-specific primers were used for RT-PCR amplification: a forward primer SD6 (5'-TCTGAGTCACCTGGACAACC-3') and a reverse primer SA2 (5'-ATCTCAGTGGTATTTGTGAGC-3'). The PCR amplification reaction was performed as follows: in 50 μL volume, 2 μl of cDNA, 5 μl of Expand High Fidelity buffer 3 (Roche, Mannheim, Germany), 1 μM of each primer, 0.8 μM dNTP_s_, and 2.6 U Expand High Fidelity enzyme mix (Roche, Mannheim, Germany) in a 9700 (Applied Biosystem, FosterCity, CA, USA) thermal cycler. Thermal conditions were 30 cycles of 95°C for 30 seconds, 58°C for 30 seconds, and 68°C for 1 minute, preceded by 2 minutes at 95°C, and followed by a final elongation step at 68°C for 10 minutes. The PCR products were separated by electrophoresis on a 3% agarose gel and each band signal was quantified by Quantity One software (Bio-Rad, Richmond, CA). All transcripts were analyzed by sequencing.

### GenBank access

The human *NF1 *exon\intron 7 sequence was derived from genomic *NF1 *sequence [GenBank: NM_000267].

## Results

Using bioinformatic identification of putative regulatory sequences and functional minigene analysis we have evaluated the effect on the *NF1 *pre-mRNA splicing process of four nucleotide variants detected in *NF1 *exon 7 of patients with a clinical diagnosis of NF1. These included a novel silent change (N335N), and three mutations (R304X, Q315Q and L316M) previously reported [22,27-29]. All variants, except R304X, were predicted by ESEfinder to destroy or create an ESE element. The predictions for the mutated exonic sequences are summarized in Table 2. The R304X mutation does not add or abolish any ESE sequence, while the Q315Q and the L316M changes abolish respectively a SF2/ASF and a SC35 ESE motif. As a consequence of the N335N change, a new ESE motif for the SRp40 protein is introduced.

**Table 2:** ESE Finder results for *NF1 *exon 7 mutations.

**Mutation**	**SRp40(1) **Threshold: 2.670		**SRp40(2) **Threshold: 2.670		**SC35 **Threshold: 2.383		**SF2/ASF **Threshold: 1.956	
Ex7 WT	3,009308		-		2,855838		2,825583	
Ex7 c.910C>T (R304X)	3,366003	↑	-		2,855838		2,825583	
Ex7 c.945G>A (Q315Q)	3,009308		-		3,281102	↑	Below Thr	↓
Ex7 c.946C>A (L316M)	3,009308		-		Below Thr	↓	3,819131	↑
Ex7 Q315Q+L316M	3,009308		-		Below Thr	↓	Below Thr	↓
Ex7 c.1005T>C (N335N)	3,009308		3,455269	+	2,855838		2,825583	

SRp40, SC35 and SF2/ASF are four human SR proteins linked to ESE sequences. ↑ indicates an increased score compared to the threshold value. ↓ indicates a score below the threshold value. + indicates the gain of a SR protein binding site.

**Figure 3:**
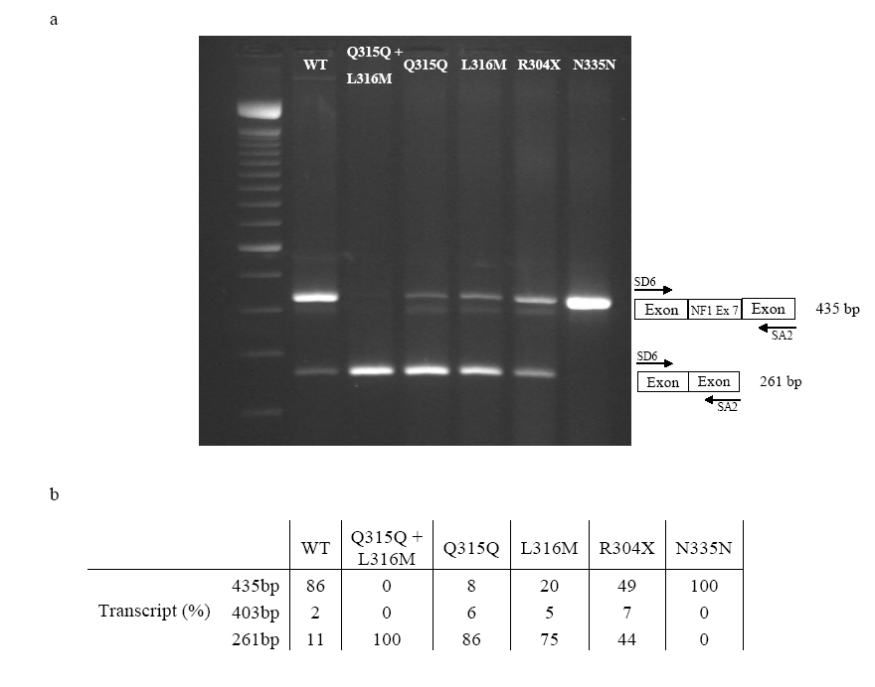
a. Post transfection PCR products of the minigene constructs obtained by SD6 and SA2 primers. The upper band indicates a 435 bp product encompassing exon 7. The lower band indicates a 261 bp product with exon 7 skipping. The central band corresponds to an alternative product of 403 bp. b. Quantification by Quantity One software of the bands showed on the top. The intensity of each band, representing the amount of each transcript, is expressed as percentage.

To confirm these predictions, we performed *in vitro *experiments that tested the splicing enhancement capacity of the wild type and mutant sequences comprising the predicted ESEs motifs. Figure 3 shows the amplified PCR spliced products of the minigene constructs, produced from primer SD6 and primer SA2 after the transfection. Each band was quantified by the Quantity One software (Bio-Rad, Richmond, CA). The wild type construct showed three splicing products in different proportions: a fragment of 435 bp (86%), another 403-bp fragment (2%), and a third of 261 bp (11%). After gel extraction, each PCR product was directly sequenced. The 435 bp PCR product included exon 7 and its intronic flanking sequences, the 403 bp fragment matched with the expected exon 7, but lacked the last 32 nucleotides, and the third band of 261 bp corresponded to a transcript lacking the entire sequence of exon 7 (NF1ΔE7). The construct containing both the Q315Q and the L316M changes showed only a single PCR product matching with NF1ΔE7. The constructs containing respectively the L316M, the Q315Q and the R304X changes disclosed the same three bands as the wild type, but in different proportions, with the NF1ΔE7 strongly represented, and corresponding respectively to 75%, 86% and 44% of the three fragments. The L316M, Q315Q and R304X constructs expressed mRNAs that included the 403-bp exon 7 product. The construct containing the N335N change showed only the 435 bp PCR product corresponding to exon 7 and its flanking intronic sequences.

## Discussion

In this study four *NF1 *exon 7 variants detected in three different patients with NF1 have been investigated using *in silico *prediction to determine their effect on ESE sequences and by *in vitro *studies to assess their functional significance on the splicing process. The mutant series included a recurrent nonsense mutation [c.910C>T (R304X)] [22,29], a novel silent change [c.1005T>C (N335N)], and a silent change and a missense mutation [c.945G>A/c.946C>A (Q315Q/L316M)] occurring together in *cis *in the same patient [27]. *In silico *analysis showed that the presence of the Q315Q and the L316M mutations each caused the loss of an ESE motif (SF2/ASF and SC35 protein respectively), suggesting a role for these nucleotide sequences in exon 7 retention in the *NF1 *message. Using the minigene assay, we demonstrated that the presence of the Q315Q and L316M mutations consistently shifted the proportion between the isoforms toward the expression of NF1ΔE7, enhancing exon 7 skipping and resulting in decreased levels of full length neurofibromin *in vivo*. Whereas the construct containing both Q315Q and L316M changes showed only the presence of NF1ΔE7 transcript, the constructs containing either the Q315Q or the L316M alone retained some wild type transcript. As predicted by ESEfinder software, Q315Q and L316M are located in two ESE motifs recognized by two different SR proteins. Each mutation individually affects only one ESE element and thus allows for the expression of a small amount of full length transcript. Conversely, the presence of both changes destroys two ESE elements and completely inhibits any residual inclusion of exon 7. According to the minigene results, RT-PCR analysis of the patient carrying the Q315Q and L316M mutations showed the presence of an additional transcript lacking NF1 exon 7, which accounts for 61% of the entire message [27]. Based on the ESEfinder results, the R304X mutation was not found to substantially affect any ESE element. However, when this mutation was placed in a heterologous minigene context, it was able to enhance the skipping of exon 7. Similar results were obtained by others expressing the R304X into a different vector [32]. Using RESCUE-ESE, a computational method alternative to ESEfinder, the same authors were able to predict that mutation R304X causes the loss of a cluster of two hexamer motifs in exon 7, but concomitantly creates two novel hexamer motifs. They speculated that the loss of the predicted ESE sites as a result of R304X has a dominant effect over the increase in the score of the new ESE sites. The results of another study suggested that R304X mutation substantially alters the predicted minimum free-energy structure of exon 7 [18], although this result has not been confirmed by others [32].

In our experiment, the search for possible SR protein binding motifs in *NF1 *exon 7 using the ESEfinder web interface [31] showed the presence of three ESE elements in the wild type sequence. This finding and the knowledge of the weak exon-intron boundaries of *NF1 *exon 7 [17,26,27] are consistent with the occurrence of exon 7 alternative splicing also seen in non-pathological conditions. Although exon 7 skipping without sequence alterations is mainly induced by stressful factors such as the amplification of RNA from aged blood [22,23], a small amount of NF1ΔE7 isoform (<1%) was also observed from RNA extracted immediately following blood drawing [17,26]. Consistently, the wild type minigene construct analysis showed the presence of three transcripts, one including exon 7, one lacking the entire exon 7 and a third lacking the last 32 nucleotides of exon 7, indicating the *in vitro *existence of an equilibrium between splicing products of the *NF1 *gene. Regarding the wild type construct, NF1ΔE7 is less expressed than the other transcripts. R304X *in silico *analysis did not produce relevant results, while *in vitro *analysis shows a reduced expression of full length transcripts, even if weaker than Q315Q and L316M mutations. The transcript lacking the last 32 nucleotides of exon 7 was expressed by all minigene constructs. To our knowledge, a messanger lacking the last 32 nucleotides of exon 7 has not been observed previously [17]. Although this transcript could represent a *NF1 *isoform expressed at a very low level, we cannot exclude that this message is an artefact of the minigene assay. In fact, the proportion of exon-including and exon lacking spliced minigene transcripts does not always correspond precisely to the levels measured in patients [32]. It should also be considered that *in vivo *a *NF1 *message lacking 32 nucleotides would result in an out-of-frame transcript, possibly degraded by nonsense-mediated mRNA decay pathway.

Whereas Q315Q, L316M and R304X mutations shift the equilibrium between *NF1 *transcripts towards the skipping of exon 7, minigene analysis of the N335N silent change demonstrated that this variant completely shifts the equilibrium between the *NF1 *messengers towards the full length transcript, being the only detectable transcript. It was previously reported that the sequence around nucleotide 1007G had a weak ESE activity and a mutation at this site caused only a mild splicing defect [32]. It was speculated that this ESE sequence was part of a composite exonic regulatory element of splicing (CERES), defined as an exonic regulatory splicing element, having overlapping enhancer and silencer functions [33]. Using ESEfinder analysis we were able to predict that the consequence of the N335N change is the introduction of a novel ESE motif for the SRp40 protein. However, ExonScan [34,35], another algorithm which simulates splicing based on known or putative splicing-related motifs, gives a different prediction showing that the mutation (c.910T>C) N335N results in the loss of an ESS (ATCAAT) motif in exon 7, but concurrently creates another novel ESS motif (AACTGG) (data not shown). As previously hypothesized for the R304X mutation and also for the N335N silent change, the loss of a putative splicing-related motif has a dominant effect over the acquisition of a new splicing-related motif. Accordingly, *in vitro *analysis has proven that nucleotide 1005T could partially belong to an ESS, since a change destroying this sequence completely eliminates NF1ΔE7 expression. The presence of some ESE/ESS elements in *NF1 *exon 7 and the existence of NF1ΔE7 in healthy individuals suggest that the splicing of exon 7 is subject to fine regulation. Notably, *NF1 *exon 7 is in-frame, thus the resulting protein could theoretically retain some function, missing only the residues encoded by this exon. As reported in our previous studies even if *NF1 *exon 7 does not belong to the neurofibromin active site it is a mutation hot spot, suggesting that exon 7 is important for the pathogenesis of NF1 [27,30,36]. It could be speculated that the NF1ΔE7 isoform could have a specific role in cellular metabolism, or that its' expression could represent a mechanism to regulate intracellular neurofibromin levels. However, the N335N change, which eliminates the *in vitro *expression of NF1ΔE7, was detected in a subject who, before clinical evaluation of full blown NF1 in his daughter and grandson, did not fit with the NF1 diagnostic criteria, manifesting only three cutaneous neurofibromas. This incomplete NF1 phenotype could be explained by the presence of undetectable mosaicism for the c.476delC mutation which was absent in the germline DNA of this subject, but was found *in cis *with the inherited N335N change in the germline DNA of his daughter and grandson. Unfortunately, none of the neurofibromas could be verified by biopsy to prove this hypothesis. Alternatively, it could be possible that c.476delC frameshift mutation could have arisen *de novo *on the same allele of N335N during spermatogenesis. However, the contribution of the N335N variant to the NF1 clinical phenotype is difficult to infer. In fact, it is difficult to imagine for this silent substitution to have the same effect on exon 7 skipping regardless of the upstream c.476delC frameshift mutation, as this type of mutations are expected to trigger NMD of the corresponding allele. However, lack of this change in normal individuals appears to rule out its polymorphic nature. Conversely, a pathogenic role for the Q315Q and L316M is easier to infer, as they are *de novo *mutations belonging to contiguous codons [27]. Moreover, as shown by the minigene analysis, the presence of each mutation individually is sufficient to enhance the expression of NF1ΔE7, thus suggesting that each of these mutations per se can result in NF1. In the present case they acted additively to cause the NF1 patient phenotype.

## Conclusion

In the present study we observed that a significant proportion of neurofibromatosis associated mutations (missense, nonsense or silent changes) residing in *NF1 *exon 7 affect pre-mRNA splicing by disrupting exonic splicing motifs, shifting the transcripts balance towards aberrantly spliced transcripts and producing two types of transcripts: those with the original mutation, and those with the skipping [27]. Therefore, it should be mandatory to investigate each nucleotide change occurring within the *NF1 *coding sequence for their potential pathogenetic role in NF1.

## Competing interests

The author(s) declare that they have no competing interests.

## Authors' contributions

IB carried out the molecular genetic studies and drafted the manuscript. ADL conceived of the study and helped to draft the manuscript. AS and VG carried out the molecular genetic studies. IT collected the samples and helped to draft the manuscript. SC and CG collected the samples. LL collected the samples and revised the manuscript. AP participated in the design of the study and revised the manuscript. BD provided overall direction to the project and revised the manuscript as well as approved the final version of it. All authors read and approved the final manuscript.

## Pre-publication history

The pre-publication history for this paper can be accessed here:


